# Air Pollutant Characterization in Tula Industrial Corridor, Central Mexico, during the MILAGRO Study

**DOI:** 10.1155/2013/521728

**Published:** 2013-01-31

**Authors:** G. Sosa, E. Vega, E. González-Avalos, V. Mora, D. López-Veneroni

**Affiliations:** Investigación y Posgrado, Instituto Mexicano del Petróleo, Eje Central Lázaro Cárdenas Núm 152 Colonia San Bartolo Atepehuacan, Delegación Gustavo A. Madero, 07730 México, DF, Mexico

## Abstract

Pollutant emissions and their contribution to local and regional air quality at the industrial area of Tula were studied during a four-week period as part of the *MILAGRO* initiative. A recurrent shallow stable layer was observed in the morning favoring air pollutants accumulation in the lower 100 m atmospheric layer. In the afternoon the mixing layer height reached 3000 m, along with a featuring low level jet which was responsible of transporting air pollutants at regional scales. Average PM_10_ at Jasso (JAS) and Tepeji (TEP) was 75.1 and 36.8 **μ**g/m^3^, respectively while average PM_2.5_ was 31.0 and 25.7 **μ**g/m^3^. JAS was highly impacted by local limestone dust, while TEP was a receptor of major sources of combustion emissions with 70% of the PM_10_ constituted by PM_2.5_. Average hourly aerosol light absorption was 22 Mm^−1^, while aerosol scattering (76 Mm^−1^) was higher compared to a rural site but much lower than at Mexico City. *δ* ^13^C values in the epiphyte *Tillandsia recurvata* show that the emission plume directly affects the SW sector of Mezquital Valley and is then constrained by a mountain range preventing its dispersion. Air pollutants may exacerbate acute and chronic adverse health effects in this region.

## 1. Introduction

Recent research initiatives in Mexico have focused on the measurement of air pollutants, with particular emphasis on ozone and suspended particles, with the objective of establishing pollution controls on most industries. Major studies in central Mexico include the *Mexico City Air Quality Research Initiative* (MARI) in 1990 [1], the *Investigacion sobre Materia Particulada y Deterioro Atmosferico-Aerosol and Visibility Evaluation Research* (IMADA-AVER) in 1997 [2], the *MCMA-2003* [3, 4], and the *Megacity Initiative: Local and Global Research Observations* (MILAGRO) in 2006. As part of the MILAGRO project, atmospheric pollutant concentrations and chemical composition were measured intensively in Tula (in the State of Hidalgo) during four weeks to determine the potential impact of contaminant emissions of Tula on the northern sector of Mexico City.

Mezquital Valley, localized some 60 km northwest of Mexico City Metropolitan Area (MCMA), is site of the Tula-Vito-Apasco industrial corridor where intensive anthropogenic activity, including oil refining, electrical generation, limestone extraction, and cement, textile and chemical production take place. In this site, both the biggest refinery in Mexico and the adjacent thermoelectric power plant use fuel oil, and it has been estimated that together they contribute with over 90% of the pollution in the valley [5, 6]. Characterization of suspended particles in the area has shown an important contribution of soil fugitive dust by agricultural activity and limestone mincing; and particle emissions from the refinery and thermoelectric power plant complex (RTPPC) covering a considerable area regardless of wind direction [6]. Additionally, the cement industry contributes with particle emissions during material extraction in open pit mines, mincing, transportation, and high temperature incineration. The SE and NE sectors of the valley are used for agriculture, most of which is irrigated with residual waters from MCMA via the Tula River. Overall, the valley is site of considerable soil, water, and air pollution. 

Several lines of evidence have associated air pollution with health effects. A direct correlation between particulate matter (PM) concentration and cardiovascular and respiratory diseases have been demonstrated [7, 8]. Recent studies have shown positive association of PM chemical composition and mortality [9, 10]. Epidemiological and toxicological studies found that the cytokine secretion patterns are influenced by the size and chemical composition of PM in Mexico City [11, 12]. A recent study evaluated the association between PM and cases of mortality, acute respiratory infections, and asthma in the Tula-Tepeji region [13]. 

The high emissions, complex topography, wind circulation, and its closeness to MCMA have prompted the monitoring and modeling of plume dispersion from the Miguel Hidalgo Oil Refinery and the Francisco Perez Rios Power Plant and their effects on the northern sector of the MCMA. In particular, it has been estimated that nearly 50% of the SO_2_ concentrations in MILAGRO Supersite T1 (NE Mexico City) are attributable to these two industries [5, 14, 15]. A similar influence of other contaminants would thus be expected. 

This paper documents a four-week daily data set on the concentrations of PM_2.5_, PM_10_, submicrometric particles, black carbon, light scattering, and criteria contaminants in addition to meteorological variables to further support the characterization of Tula emissions as a potential source of pollutants to Mexico City and nearby cities (Tepeji and Jasso). In addition, stable carbon isotopes in the epiphyte *Tillandsia recurvata* collected in Mezquital Valley during the study are used here as a proxy of the long-term trajectory of the emission plume.

## 2. Study Area and Experimental

### 2.1. Sampling Sites and Measurements

The Miguel Hidalgo refinery is the second largest refinery in Mexico, processing 3 × 10^5^ barrels of crude oil per day, and it supplies the 50 × 10^6^ liters per day of gasoline-equivalent (all fuels included) to Mexico City [16]. In turn, the Francisco Pérez Ríos power plant has 1.5 GW of capacity and it mainly supplies the electricity demand for Central Mexico. The power plant has four major units fueled primarily by fuel oil (4% sulfur by weight) and five small combined cycle units burning natural gas. In comparison, urban emissions due to transport and service/commerce activities in nearby localities (Tula de Allende, Atotonilco, Atitalaquia and Tepeji) represent a small contribution to local air pollution [17].

Three sampling sites within the Tula-Vito-Apasco corridor were selected to measure PM, and upper and surface meteorological parameters (Table 1). 

#### 2.1.1. Tula (TUL) Upper Air Monitoring Station

Tula (TUL) located inside the Instituto Mexicano del Petróleo facilities adjacent to the refinery (99.27°W, 20.05°N). This site was selected as an upper air monitoring station to determine the diurnal evolution of the mixing height (MH) as well as other meteorological variables, such as wind direction (WD) and wind speed (WS). These variables are essential in air quality studies to determine the vertical extension in which pollutants are mixed, dispersed, and transported [18].

A Vaisala radiosonde system (Model SPS-220) was used for upper air sounding measurements of pressure, temperature, relative humidity, and the horizontal wind vector, at different altitudes. Vertical profiles were determined by launching four rawinsondes everyday at 08:00, 12:00, 15:00, and 18:00 h CST. Additionally, surface meteorological parameters, temperature (*T*), ambient pressure (*P*), relative humidity (RH), wind direction (WD), wind speed (WS), and solar radiation (SR) were also measured using a regular meteorological station. These measurements took place from 18 March to 17 April, 2006. 

#### 2.1.2. Jasso (JAS) and Tepeji (TEP) Surface Stations

JAS and TEP were considered as core sites where several equipments were installed to simultaneously measure criteria pollutants (CO, NO_2_, O_3_, and SO_2_), PM, and submicrometric particles. These sites are influenced by both urban and major industrial sources. JAS (99.31°W, 20.02°N) is located about 5 km SW from the refinery and the power plant, whereas major urban intercounty heavy traffic roads are located south and west. This site is close to a major limestone area where mining for cement materials occurs. TEP (99.29°W, 19.86°N) is located 21 km S away from the refinery, far from urban areas but close to a major highway. This site is also close to the major cement plant and near limestone mining areas.

### 2.2. Meteorology and Criteria Pollutants

Surface meteorological parameters and criteria pollutants were measured from 22 March to 21 April 2006 at TEP, and from 25 March to 22 April at JAS. Measured variables included temperature, pressure, relative humidity, wind speed and wind direction, and SR (*T*, *P*, RH, WS, WD, and solar radiation, resp.). Criteria pollutants were measured using a mobile laboratory equipped with conventional analyzers (Monitor Labs). Methods used to determine criteria pollutants were NOM-034-SEMARNAT-1993 using dispersive spectroscopy for CO, NOM-037-SEMARNAT-1993 using chemoluminescence for NO_*x*_, USEPA-EQOA-0193-091 using UV photometry for O_3_, and USEPA–EQSA-0193-092 using pulse fluorescence for SO_2_. 

### 2.3. Particulate Matter (PM)

PM samples were collected daily from 00:00 to 24:00 h using portable low volume MiniVol samplers (Airmetrics, Springfield, USA) at a flow rate of 5 L/min, previously calibrated at standard conditions. In addition sequential filter samplers (SFS) equipped with PM_2.5_ (Bendix 240 cyclones) and PM_10_ (Andersen SA254) samplers operated at a flow rate of 113 L/min were used to collect 12-hour samples (06:00 to 18:00 and from 18:00 to 06:00). Samples were collected on 47 mm Teflon-membrane (Pall Gelman Laboratory, Ann Arbor, MI, USA) and quartz-fiber filters (Pallflex Gelman Sciences CT, USA). Teflon filters were used for mass, trace element analyses, and for light transmission, whereas quartz filters were used for ion and elemental and organic carbon analyses [19].

### 2.4. Submicrometric Particles and Optical Properties

Light scattering coefficients were measured with an integrating nephelometer (Model 3563 TSI, Inc.) operating at 450, 550, and 700 nm. Submicrometric particle surface distribution was obtained with a scanning mobility particle sizer (SMPS) (Model 3936 TSI, Inc.). Black carbon mass concentrations were measured with an aethalometer (Model AE-16 Magee Scientific, Co) operating at 880 nm.

### 2.5. Stable Carbon Isotopes

Long-term atmospheric deposition patterns for selected trace metals, PAHs, and stable carbon isotopes covering an area of 4000 km^2^ were determined with samples of ball-moss (*Tillandsia recurvata*) at 50 sites as atmospheric biomonitors. These patterns reflect the long-term air pollutant transport from the oil industry and other economic activities in Mezquital Valley according to the prevailing winds. This moss satisfies basic attributes of a biomonitoring organism, such as its widespread occurrence in the valley, and its dependence on atmospheric nutrients and humidity [20, 21]. Therefore metal, PAHs, and stable carbon isotope composition in this species serve as proxies of atmospheric emissions impact at different distances from the emission source. In this paper, stable carbon isotopes in *T. recurvata* were used as a tracer of long-term signature of the plume trajectory. Metals, stable isotopes, and PAHs in *T. recurvata* are described by Zambrano García et al. [22] to show the signature source of the principal emitters in Mezquital Valley.

Total flux emissions and tracking of the SO_2_ plume were determined at major sources in the region to identify transport pathways towards Mexico City under different meteorological conditions using a mobile Differential Optical Absorption Spectroscopy (MiniDOAS) system. Details of the technique and results are described by Rivera et al. [5].

**Figure 1:**
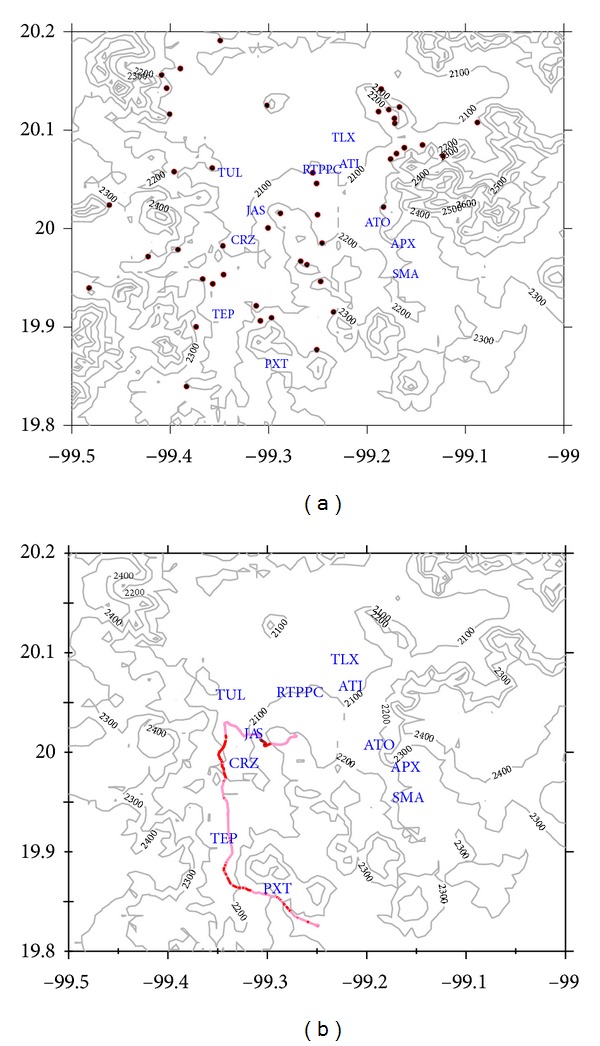
(a) Mezquital Valley topography with sampling sites (circles), major population centers, and the refinery-thermoelectrical plant complex. (b) Trajectory of the SO_2_ dispersion plume in SE direction observed on April 4, 2006 at 16:00 and 17:00 hours. APX: Apaxco, ATO: Atoyac, ATI: Atitalaquia, CRZ: Cruz Azul, JAS: Jasso; PXT: PEMEX gas substation; RTPPC: refinery thermoelectrical power plant complex; SMA: Santa María Apaxco; TEP: Tepeji; TLX: Tlaxcoapan; TUL: Tula.

Figure 1 shows the sites where *T. recurvata* were sampled in the vicinity of the refinery and thermoelectric power plant complex (RTPPC), which is located nearby several small towns, at an altitude of around 2100 m above sea level. Note the mountain chain to the E-SE of the RTPPC which rises approximately 400 m above the valley. Winds measured in March to April, 2006 [22], and October to December, 2008 [23] in the region show highest speeds when coming from the NE and SW quadrants. Figure shows the SO_2_ concentrations measured along the miniDOAS path.

## 3. Results and Discussion

### 3.1. Meteorology

#### 3.1.1. Synoptic Overview

Field measurements and large-scale analysis showed that local meteorological conditions and air pollutants transport were affected by the synoptic systems development. Detailed descriptions on meteorological conditions during the MILAGRO campaign are reported by Fast et al. [24]. Three major synoptic-scale systems were observed during the field campaign. The more frequent events were high-pressure systems, remaining quasi-stationary at central Mexico, followed by troughs and rides passages at south-central U. S. Cold surges. The third and least frequent system was cold surges (northern) influencing mainly the coast line at Gulf of Mexico and was also present during the study period. Both during early March and on March 18-19, upper-level troughs propagated through south-central U. S. producing strong southwesterly winds in the central plateau. Two cold surges were observed on March 22 and 24–26 causing strong northerly near-surface flow over the Gulf of Mexico. High-pressure systems predominated during the remaining sampling days, and were typical characterized by clear skies, low humidity, and weak winds. 

Winds below the mixing layer at 700 hPa (1000 m above ground level) were variable according to the dominant synoptic system in the region. Westerly and southwesterly winds were associated to a trough pass; northerly and easterly winds to high-pressure systems slowly moving from northwestern Mexico towards the east, and strong northerly near-surface flows over de Gulf of Mexico were associated to cold surges. The frequency of upper wind direction at 500 hPa measured at 06:00 h were 45.2% southwesterly, 35% westerly, 13% northeasterly, and 3.2% corresponding to south and northwesterly winds. Daily maximum surface temperature was over 22°C most of the days; however, during the period of March 24–26, during a cold surge passage, temperature dropped to 18°C. Surface wind speeds, varying from light to moderate, were observed throughout the study.

#### 3.1.2. Height of the Boundary Layer

**Figure 2:**
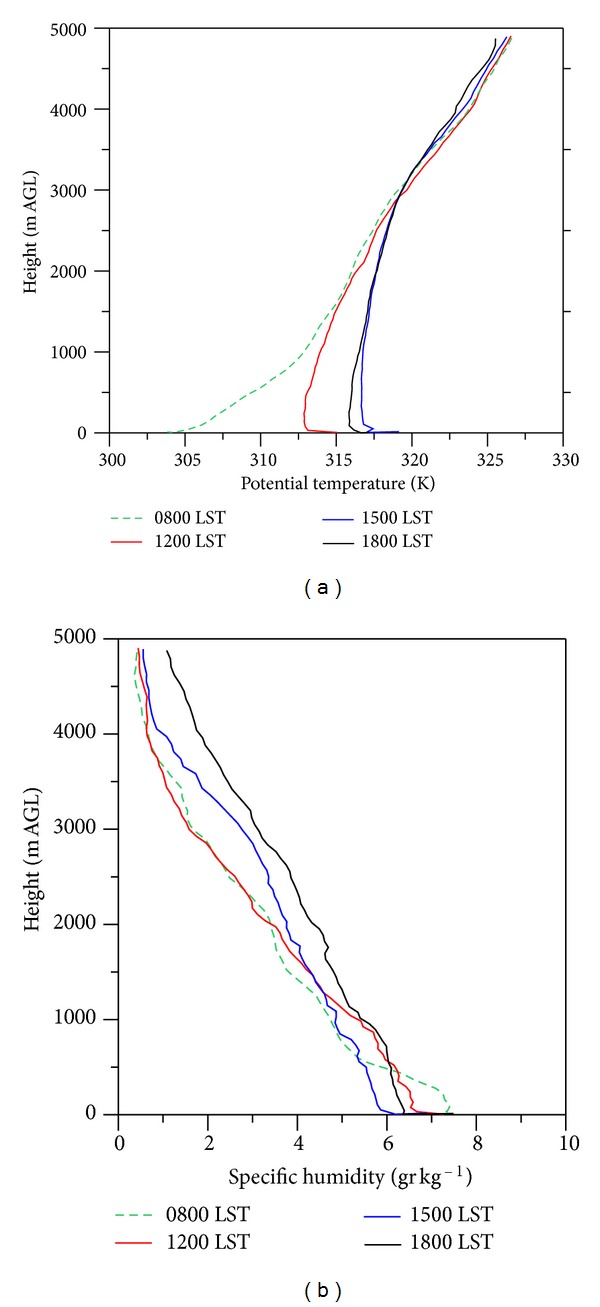
Mean potential temperature (a) and specific humidity profiles (b) at Tula.

Figure 2 shows the mean potential temperature and specific humidity profiles for the field campaign. The profiles were obtained averaging all radiosonde data available at each launch time. During most of the sampling period (80% of the time), a stable layer was observed at 08:00 h, with the occasional presence of near-surface thermal inversions; by noon, when solar heating breaks the stable layer, a well-developed mixing layer reached up to 2000 m above ground level (AGL), with a shallow superadiabatic layer near the surface. Although potential temperature profiles were similar above 500 m at 15:00 and 18:00 h, the colder temperatures at 18:00 h, indicated the formation of a stable layer close to the surface (Figure 2(a)). Specific humidity profiles showed a reduction of water content with height, and a well-mixed atmosphere after the breakup of the stable layer. The difference on the water vapor content among vertical profiles was less than 4.0 g/Kg throughout the study (Figure 2(b)). 

Data analysis of the radiosonde data showed that the mixing layer height (ML) varied significantly from 2500 to 4200 m AGL, reaching its average diurnal maximum at 15:00 h. When a strong cold surge entered Central Mexico during March 24-25, the ML decreased to less than 500 m AGL, due to a reduction in the solar radiation as a consequence of cloudy skies and scattered rain.

#### 3.1.3. Mean Wind Profiles at the Boundary Layer

**Figure 3:**
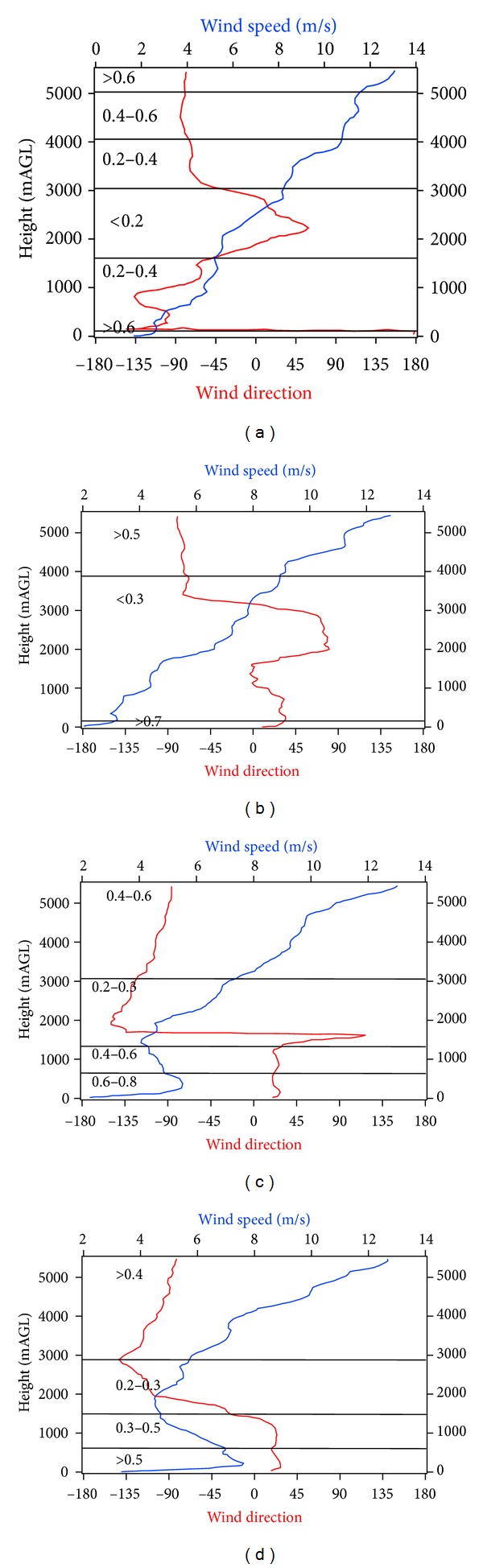
Mean wind profiles at Tula, during March-April, 2006. Horizontal lines represent the wind persistence value.

Figure 3 gives the mean wind profiles calculated from rawinsondes data during the field campaign at 08:00, 12:00, 15:00, and 18:00 hs. The horizontal lines represent variations with height of the wind persistence, defined as the ratio of the vectorial mean wind speed to scalar wind speed. Ratios close to one indicate almost no change in wind direction; on the contrary, values close to zero indicate a large variation in wind direction. 

Figure 3(a) shows a highly variable and stratified atmosphere at 08:00 h. The change in wind persistence with height varied from less than 0.2 to greater than 0.6, being more stable at heights lower than 100 m AGL and above 5000 m AGL. Near-surface southerly to southwesterly winds were observed regularly along the field campaign. At 12:00 h, a less stratified atmosphere was observed, although a varying wind speed layer, from 300 m to 4000 m AGL, still remained (Figure 3(b)). Below 1500 m AGL, northeasterly wind speed was more stable; however, wind speed increased constantly and therefore the wind persistence was low, reflecting the ML evolution into a well-mixed layer. At 15:00 h, the wind persistence ratio ranged from 0.2 to 0.86 under the lowest 5000 meters of the troposphere, although a clearly more homogeneous mixing layer was observed (Figure 3(c)). Below 1500 m, the mean northeasterly wind remained constant, and the wind speed profile shows a northeasterly jet near the surface, with a maximum value close to 6 m/s at 500 m AGL.  Figure 3(d) shows the mean wind profile at 18:00 h, which is similar to the wind profile at 15:00 h, but with more variability in wind direction and with a stronger jet closer to the ground (at 250 m and maximum velocity of 7 m/s).

In summary, early in the morning (8:00 h), a shallow stable layer formed near the surface, with light south-southwesterly winds. These conditions favored the accumulation of air pollutants below 100 m with a limited dispersion towards the north Tula industrial complex. After midday, a well-developed mixing layer favored the mixing of pollutants at higher altitudes (up to 5000 m AGL). Simultaneously, a near-surface superadiabatic layer, with a clear northern wind component, brought back the previously-advected pollutants. In the afternoon and evening a thicker mixing layer reached on average 2500 to 3000 m height, featuring a weak low level jet (LLJ) near the surface. This LLJ was responsible of transporting air pollutants at regional scales. According to the authors' knowledge, this is the first time this feature is reported for the region. 

LLJ can develop under favorable synoptic conditions anywhere in the world. This pattern is characterized by space scales between 20 and 200 km within the lowest 2000 m of the atmosphere, where strong diurnal oscillations and nocturnal accelerations occur [25]. The development of LLJ is associated with nocturnal temperature inversion, forming in the late afternoon or early evening and becoming strongest during the early morning hours of the next day, and then weakening or disappearing by late morning. From Figures 2(a), 3(c), and 3(d), it is clear that temperature and wind profiles fulfill these requirements. Although no early-morning sounding was available to corroborate that, maximum wind speed occurred at this time.

Implications of these meteorological conditions on human health are evident. First, the persistent stable atmospheric conditions in the morning favor the concentration of pollutants near ground, increasing the level of exposure of the inhabitants. This condition may worsen at winter time, when lowest temperatures drop below 0°C. On the other hand, the regional transport of pollutants increases the number of people exposed to these air pollutants. These results are in agreement with those recently reported by Almanza et al. [15]. By using dispersion models, the authors estimated the fraction of time at which the RTPPC plume hits the surface in the full domain. Regardless the concentration at which the plume reaches the ground, the impact on the Mexico MCMA may be 40% to 60% of the time [15]. 

### 3.2. Criteria Pollutants

Air quality standards (AQS) at JAS such as ozone (0.11 ppm, average of 1 h), carbon monoxide (11 ppm mobile average of 8 h), sulfur dioxide (0.13 ppm average of 24 h), and nitrogen dioxide (0.210 ppm, average of 1 h) were not exceeded [26]. Maximum hourly values were 0.034, 0.033, and 0.287 ppm for O_3_, NO_2_, and CO, respectively. The maximum average of 24 h for SO_2_ was 0.0078 ppm, on April 5, even though this pollutant increased to 0.293 ppm at midday on April 14.

The results at TEP showed that CO and NO_2_ concentrations were below their corresponding AQS values. However, the SO_2_ exceeded the standard twice, on March 26 with 0.185 ppm, and April 14 with 0.190 ppm. O_3_ also exceeded the standard during the April 27 with 0.122 ppm. In general, this site showed higher concentrations of SO_2_ when compared to JAS, with maximum hourly values between 0.100 and 0.300 ppm.

Most of the SO_2_ is released from the refinery and the RTPPC facilities at a height of 60 m above ground level, although the effective height of the emissions may rise above 1000 m, due to buoyancy provided by hot exhaust. Under these conditions, the emission plume travels long distances downwind before reaching the ground, which is in agreement with mini-DOAS measurements reported by Rivera et al. [5]. Sites located nearby the source are below the plume and thus report lower SO_2_ concentrations when compared to those farther from the plume source (Figure 1(b)).

### 3.3. Particulate Matter (PM)

Table 2 summarizes the PM10 24 h average concentrations during the sampling period for March 24-April 20, 2006. Also the 24 h average PM_2.5_ concentrations during March 24-April 6, 2006 and 12 h average PM_2.5_ concentrations during April 7–20, 2006 were given at standard conditions. In general, PM concentrations measured at the JAS urban-industrial site were higher than at TEP, which may indicate the magnitude of local limestone dust resuspension from quarrying. Particle contribution at this site is exacerbated by the cement industry which is also a heavy user of residual fuel oil, petroleum coke, and an assortment of industrial wastes.

PM mass concentrations oscillated substantially from day to day at both sites. The most extreme variations occurred at JAS in the PM_10_ fraction and PM_10_ levels at JAS showed considerably variations from 30.4 to 178.6 *μ*g/m^3^ with an average of 75.1 *μ*g/m^3^; the 24 h average of 120 *μ*g/m^3^ [27] was exceed twice during the study. While PM_2.5_ ranged between 14.3 to 52.0 *μ*g/m^3^ with an average of 31.0 *μ*g/m^3^, the PM_2.5_ standard of 65 *μ*m/m^3^ was not exceeded during the sampling campaign. High PM_10_ concentrations were observed on April 8 and 11 with values of 162.2 and 178.6 *μ*g/m^3^, respectively. Those two days had light wind speed in the morning (<1.56 m/s) with variable wind direction. After 10:00 h, southwest winds became more stable and increased their speeds to a maximum of 8.0 m/s at 18:00 h. On these days, the corresponding PM_2.5_ mass concentrations (49.5 and 36.7 *μ*g/m^3^, resp.) were less than one-third of the PM_10_, which is less than the PM_2.5_/PM_10_ average ratio of 0.41, and suggests that elevated PM_10_ concentrations can be attributed to local fugitive dust. Similar temporal variations of PM_2.5_ mass concentrations were observed from March 26 to April 3 with a smooth increase from and with two peaks on the same dates as PM10. Similar PM10 levels were reported by Querol et al. [28] in the urban area of Mexico City with 24-hour averages ranging between 50 and 56 *μ*g/m^3^, and PM_2.5_ between 24 and 40 *μ*g/m^3^; however, these values were measured during March as a part of the MILAGRO campaign and cannot be compared directly with our observations.

At TEP PM_10_ concentrations were higher during a longer period of time (April 5–13). In general, during April winds were light (2 m/s) and variable in average, although maximum daily speeds ranged from 7 to 9 m/s. During April 5–13, higher wind speeds were recorded compared to the period of April 14 to 19, where lower PM concentrations were observed, due to more stable wind conditions. Wind directions were variable before 8:00 h, were persistent from southeast until noon, and changed direction from northeast in the evening. PM_10_ concentrations at TEP ranged from 22.4 to 53.2 *μ*g/m^3^ with an average of 36.8 *μ*g/m^3^, while PM_2.5_ varied between 14.5 to 53.1 *μ*g/m^3^ with an average of 25.7 *μ*g/m^3^. Although the fine fraction at both sites were similar, the difference was driven by the PM_10_ fraction as the PM_2.5_/PM_10_ mass ratio ranged from 0.85 to 0.50 with an average of 0.68 indicating that this site is a receptor of the industrial emissions from the major sources of combustion processes. 

The PM_2.5_/PM_10_ ratio at JAS is similar to those reported by Vega et al. [19] for Mexico City (0.46), and for the industrialized area of Salamanca (0.33) in Guanajuato [29], where the contribution of fugitive dust is considerable.

PM_2.5_ was consistently higher during the morning periods (06:00–18:00h), with average values of 35.5 and 45.5 *μ*g/m^3^, and decreased during the night (18:00–06:00 h) to 24.9 and 31.0 *μ*g/m^3^ at TEP and JAS, respectively. This is consistent with a recurrent shallow stable layer formed near the surface before 8:00 h and light winds favoring particle accumulation. At TEP maximum concentrations of 75.1 and 37.6 *μ*g/m^3^ occurred in April 11 and 16 for morning and night, respectively. In contrast, JAS maximum concentrations were measured in the morning of April 8 (70.3 *μ*g/m^3^) and at night on April 7 (59.2 *μ*g/m^3^).

**Figure 4:**
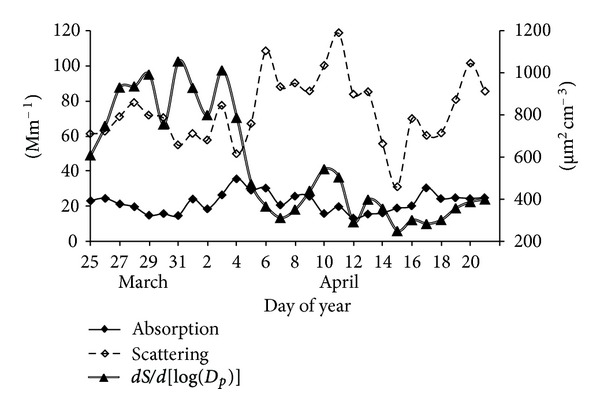
Daily aerosol light absorption and scattering obtained at 550 nm, and submicrometric particles surface distribution, at JAS from March 25 to April 21, 2006.

The aforementioned pattern is in agreement with the continuous black carbon measurements which were influenced by fresh emissions from major industrial combustion sources (Figures 4 and 7). Similar patterns were also reported for Mexico City during the same period of sampling [28]. It should be mentioned that carbonaceous aerosols, specifically black carbon, are the major absorbing aerosol species which may have an important warming impact at a regional scale. The aethalometer used to measure black carbon takes into account the flow rate, time, and a factor for the multiple scattering enhanced mass absorption efficiency [30]. 

There was a significant difference between both sites in the coarse fraction (PM_10_-PM_2.5_), JAS being the mostly impacted by particles resulting from local limestone dust from quarrying, as it ranged from 40 to 79% with an average of 56%. At TEP this fraction varied from 15 to 50% with an average of 32%,. These results are consistent with higher emission from fossil fuel combustion processes from the RTPPC), among other sources in the vicinity of TEP (21 km south from the sources). In turn, Querol et al. [28] reported 50% of coarse fraction at CENICA, similar to JAS, while the urban T0 site had a lower contribution of coarse fraction (30%), similar to TEP.

The incidence of respiratory diseases is higher in Tula than in any other area within Hidalgo State. Specifically, the central area of Tula the incidence of acute respiratory infections is the highest within Mexico [31]. In addition, Melgar-Paniagua et al. [13] reported that there is an association between PM concentration and the increase of respiratory morbidity and mortality in Tula. Furthermore, there is evidence that exposure to ambient PM chemical components, such as sulfate, metals, elemental, and organic carbon, is associated with adverse health outcomes [32, 33].

Results from chemical analysis, which will be presented in a future publication, showed that at JAS the higher concentrations of PM_10_ were driven by Ca, whilst in Mexico City the higher PM_10_ concentrations were correlated with Si [19]. High Ca concentrations are mainly due to limestone mining for cement materials and limestone quarries nearby JAS and in the surroundings of Tula [22]. This contrasts with Si-associated PM_10_, which result from dust resuspension from the dry Texcoco Lake in Mexico City [19]. Higher Ca concentrations and in some cases also high SO_4_^=^ concentrations were also correlated with PM_2.5_. Sulfates showed a gradually increase at TEP starting on April 9 and showed a peak on April 12. The results of the radiosonde data analysis, below 1000 m which is the maximum mixing layer height during the day, confirm that on April 12, there were persistent winds all day coming from north and northeast with an average velocity ranging from 4 to 9 m/s with direct influence from limestone quarries located upwind. On the other hand, Ca generally showed a similar pattern to PM_10_ mass with peaks driven by high Ca concentrations. On April 16, however, low Ca concentrations and high PM_10_ values were observed. A deeper mixing layer was developed on that day, reaching 3.5 km. Winds were lighter and its direction changed early in the morning from south to west at noon, and then in the afternoon from north to east in the evening. This suggests that different emission sources contributed to high particle concentration, including those from agricultural activities, where Si is abundant.

### 3.4. Optical Properties

Light scattering, absorption, and surface size distribution represent different physical properties of particles and no direct comparison among them is always possible; however, all these parameters are a function of particle size and an analysis of its behavior can give an insight from the origin of particles.

#### 3.4.1. Light Scattering

Figure 4 shows the daily light scattering distribution determined with a nephelometer at 550 nm. In general, a large variability was observed along the sampling period with high light dispersion episodes observed on April 6–8 and April 11–13 when low mixing heights were observed. After April 15 the scattering values decreased to background values. Similar trends for 450 and 700 nm wavelengths were observed (not shown). This high dispersion associated with low mixing heights is consistent with pollutant concentration near the surface. In contrast, a decrease in light scattering, and thus a greater visibility occurred when mixing heights were greater.

Since Tula is classified as a “critical area” due to the high SO_2_ and PM emissions, it should also be expected to have high SO_4_^=^ concentration. Sulfate is an important light scattering aerosol species contributing to atmospheric cooling, formed from the atmospheric oxidation of SO_2_ [30].

**Figure 5:**
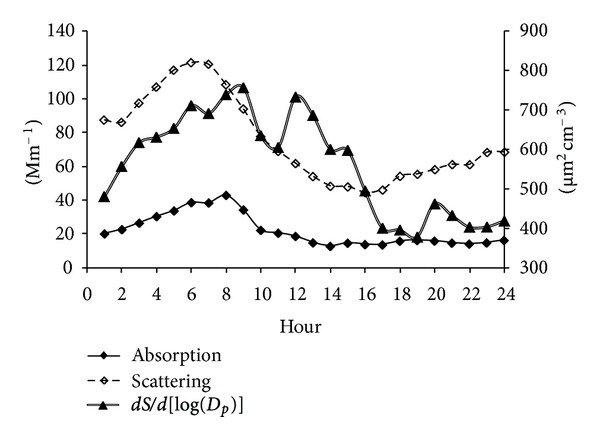
Hourly aerosol light absorption and scattering obtained at 550 nm; submicrometric particles surface distribution at JAS from March 25 to April 21, 2006.

The hourly aerosol scattering at JAS, at 550 nm ranged from 44–121 Mm^−1^ with an average of 76 Mm^−1^. When compared to the rural site of Tecamac (53 Mm^−1^) located 29 Km northeast of Mexico City, this value was higher by a factor of 1.4, but much lower than the urban area of Mexico City (105 Mm^−1^) reported by Marley et al. [34] during March 10–29, 2006. Light scattering is clearly an important factor during the night and before 10:00 h, with a substantial reduction afterwards (Figure 5). 

Higher scattering values, due to a persistent shallow stable atmospheric layer which traps most primary pollutants emitted below 500 m (Figure 2), were observed at night and before 10:00 in the morning, reaching a maximum at 7:00 with values as high as 121 Mm^−1^, 3.5 hours earlier than the maximum scattering values seen at Mexico City [34] suggesting a rapid secondary aerosol formation. After 10:00 h, a decrease of scattering values was observed due to the development of the mixing layer favoring the dilution of air pollutants.

#### 3.4.2. Submicrometric Particles

**Figure 6:**
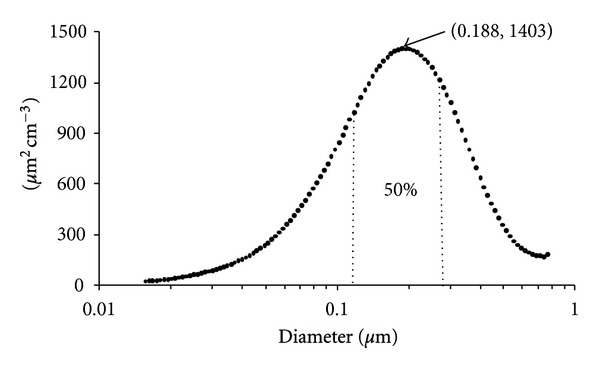
Submicrometric particles surface distribution by size, at JAS from March 25 to April 21, 2006.

Figures 4–6 show the submicrometric particle surface size distribution (dS/d[log(*D*_p_)]), calculated by the SMPS system assuming spherical particles with diameters (*D*_p_) from 15.7 to 764 nm, measured at JAS from March 24 to April 21, 2006. In general, highest values were observed during March 26 to April 5 (Figure 4). The hourly highest values were observed between 6:00 and 9:00 h and after noontime (12:00 to 15:00 h) (Figure 5). Particles with diameters between 0.118 and 0.269 *μ*m contributed with 50% of the total particle surface size distribution. Particles smaller than 0.118 *μ*m and higher than 0.269 *μ*m contributed with 25% each (Figure 6). Implications of exposure to these particles on human health are presented by Buonanno et al. [35, 36], which studied average particle number size distribution data together with the people activity pattern to estimate the tracheobronchial and alveolar dose of submicrometer particles for different population age groups and under different exposure microenvironments in Italy. 

#### 3.4.3. Black Carbon

An absorption Angstrom exponent of 1 [34–38] was applied to the calculation of attenuation to determine its dependence with wavelength. Since almost all fresh emissions in this region come from the combustion of fossil fuel at the refinery and the power plant then the specific attenuation of 14625/*λ* [37] was used to convert black carbon concentrations measured at 880 nm and be able to compare to results of aerosol absorption at 550 nm measured at the same period reported by Marley et al. [34].

**Figure 7:**
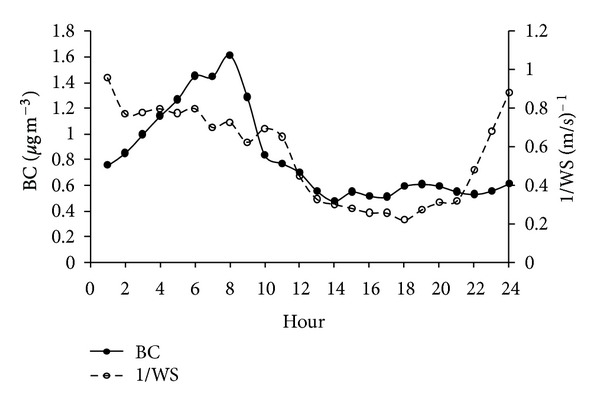
Hourly average black carbon (BC) concentration and reciprocal wind speed (1/WS).

Figure 7 shows that the highest concentration of BC from 2:00 to 11:00 h is coincident with the reciprocal of wind speed (1/WS) peak. There is an increase of 10.3%/h of BC concentrations from 1:00 to 8:00 h, and a decrease of 4.2%/h of the 1/WS, suggesting that fresh emissions have a major effect than the light wind speed (1.36 to 1.85 ms^−1^); furthermore, the boundary layer was stable favoring stagnation of pollutants. From 09:00 to 18:00 h, both BC and 1/WS decrease at a similar rate of 15 and 12%/h, respectively. During this period the mixing layer is more important than either the fresh emissions or the wind speed. From 19:00 to 24:00 h, the air parcel stagnation increased 22%/h meanwhile BC increased only 0.6%/h, so neither the fresh emissions nor the wind speed or the mixing layer were dominant for determining the levels of BC.

Figures 4 and 5 show the daily and hourly aerosol light absorption measured at 880 nm and corrected to 550 nm at JAS from March 24 to April 21, 2006. In general, there was a continuous increase during the first hours of the day, from 01:00 to 08:00 h, and then there was a decrease from 9:00 to 14:00 and from 15:00 to 24:00 h, there was an average absorption of 15 Mm^−1^. On a daily basis, highest absorptions were observed during April 4–6 with up to 36 Mm^−1^ (Figure 4) which correlates with an excess of liquid products flaring in the refinery due to a failure in a process that lasted two days. The highest absorption reached 43 Mm^−1^ between 2:00 and 9:00 h (Figure 5). The hourly aerosol light absorption varied between 13–43 Mm^−1^ with an average of 22 Mm^−1^ which is smaller compared to both values at Mexico City (37Mm^−1^) and Tecamac (27 m^−1^) reported by Marley et al., [34]. The lower light absorption at Tula compared to both sites results from the single point source behavior of the industrial complex; therefore the plume sweeps the region out according to local winds, and not always reaching the monitoring device. In addition, Marley et al. [39] observed large amounts of biomass burning contributing to carbonaceous aerosols at Tecamac and that Mexico City was significantly impacted by emissions from fossil fuels in addition to local and regional burning. 

### 3.5. Tula-Vito-Apasco Industrial Corridor SO_2_ Export

Nearly 20 plume transects were measured with a 3 to 8 h time-spam, for an equivalent distance of 120 to 320 km. The SO_2_ plume direction was variable during the period of study, mostly affected by meteorology at synoptic scale. The plume's most frequent direction was from southeast after midday, when the wind at the surface was from NNE. Under this condition, the SO_2_ plume did cross the Mexico-Queretaro highway, SW of the Tula-Vito-Apasco corridor. Other less frequent episodes showed the SO_2_ dispersion towards the east of the industrial area, which after interacting with the mountain range diminishes the transport of the SO_2_ plume outside the area.

Figure 1(b) shows the trajectory of the SO_2_ dispersion plume observed on April 4, at 16:00 and 17:00 h. Two routes that were selected to follow the SO_2_ plume. The red colored lines show the relative SO_2_ column concentration measured using the MiniDOAS system. Route 1 was all around the refinery through the southeast. Route 2 includes a north-south transect from Tula City to Tepeji. The SO_2_ plume is dispersed along routes 1 and 2 in the southwest direction. The aforementioned dispersion trajectory was the most common, although other less frequent trajectories were also observed. For instance on April 7, the SO_2_ plume dispersed through the north and then at midday to the southeast (not shown).

**Figure 8:**
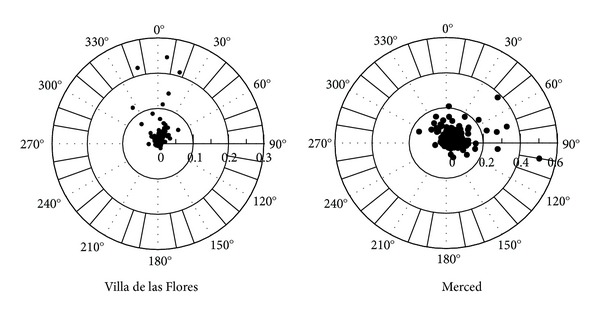
Wind roses of the SO_2_ concentrations (ppm) for the Villa de las Flores (VIF) and La Merced (MER) stations from 18 of March and 17 of April.

On the other hand, wind roses (Figure 8) of the SO_2_ concentrations (ppm) were drawn from March 18 and April 17 for the Villa de las Flores (VIF) and La Merced (MER) stations, which are located most north and downtown of Mexico City, respectively. The results showed that highest SO_2_ concentrations (0.10 to 0.25 ppm) at VIF were associated to northerly wind flows (330°–30°). On the other hand, at MER the SO_2_ concentrations were 25% lower than those observed at VIF, and the highest values (>0.02 ppm) occurred when winds flows were from the NE-E (45°–105°). These results suggest that SO_2_ emissions from Mezquital Valley have an impact on the northern sector of MCMA. In turn, SO_2_ concentrations were higher at VIF compared to those measured at JAS (0.13 ppm), implying that the height of the emitted plume from the RTPPC has a higher impact outside the emission point. This is in agreement with model simulations for Tula reported by de Foy et al. [40].

### 3.6. Long-Term Plume Trajectory

**Figure 9:**
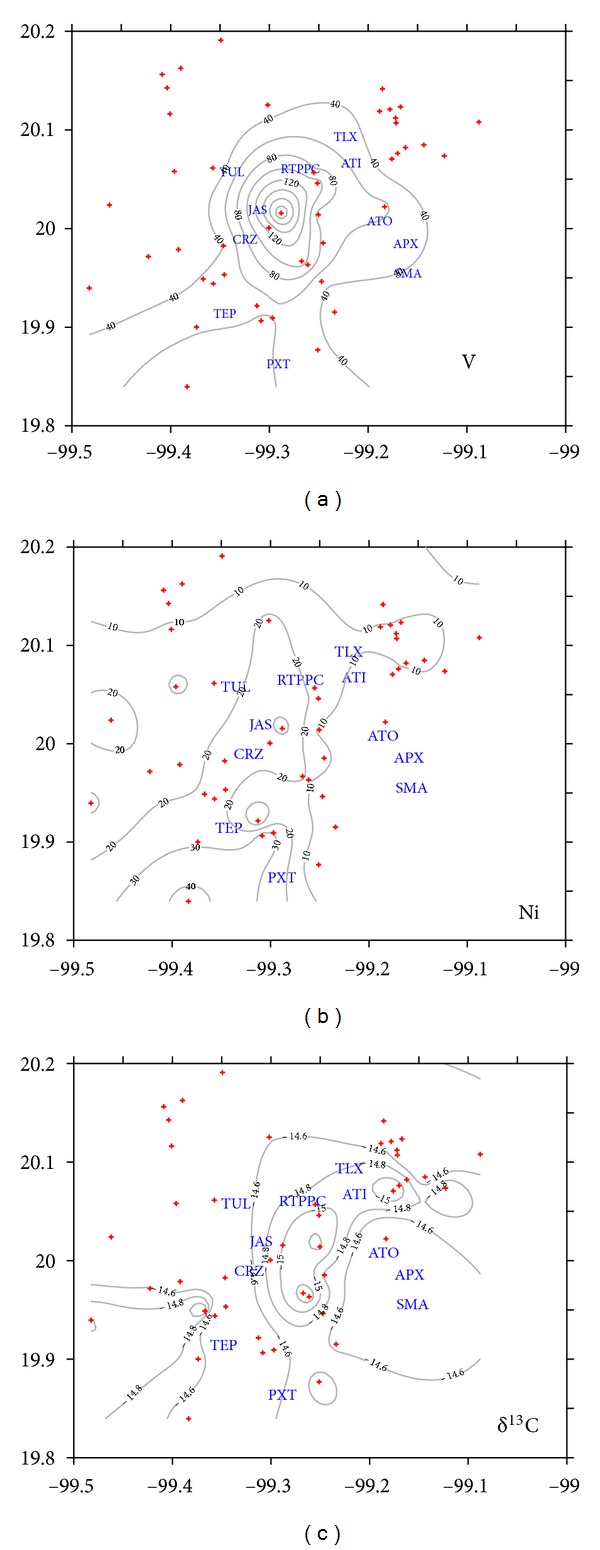
Nickel and vanadium concentrations (in mg/kg) and stable carbon isotope composition (in permile versus PDB) in *Tillandsia recurvata* samples collected in Mezquital Valley.

Figure 9 shows the emission plume that develops from the RTPPC as depicted by the higher vanadium and nickel concentrations and ^13^C-depleted carbon values in *T. recurvata* in Mezquital Valley relative to background. These metal concentrations and carbon isotope composition represent the long-term pattern area of influence of the emissions from the complex and show that it impacts those towns located along a NE-SW axis between Tlaxcoapan and TEP, and further exits out the valley. The highest nickel and vanadium concentrations in *T. recurvata* were equally distributed at both sides of the axis. In contrast, *δ* ^13^C values show lighter values to the east of this axis. Metals are likely emitted with particles and would tend to disperse equally at both sides of the long-wind axis, settling within a relatively short distance from the emitter. In contrast, carbon would be mostly emitted as CO_2_ and assimilated as such by the epiphyte. It thus appears that gas emissions are influenced by a mountain effect: when wind blows from the SE quadrant the plume disperses into the valley and dilutes the emitted CO_2_. In contrast, when the wind direction is from the NW or NE quadrants, emissions tend to concentrate between the RTPPC and the mountain chain located to the S-SE of the complex. Therefore, the relatively longer residence time of the emissions under NW or NE quadrant winds lowers the ambient carbon isotope composition of CO_2_. 

The relative contribution of CO_2_ emitted by the RTPPC can be estimated from the average *δ* ^13^C value of the epiphyte. The carbon isotope composition of *T. recurvata* decreased from an average of −14.6‰  outside the emitted plume to −15.4‰  inside the plume. This 1.8‰  decrease suggests that industrial emissions contribute with 5% of the total CO_2_ incorporated by *T. recurvata* inside the plume. By stable isotope-mass balance, it follows that industrial CO_2_ has a *δ* ^13^C value of −30.0*‰*, which is consistent with the range of −29.5 to −27.6‰  for CO_2_ emitted for the combustion of diesel and fuel oil [41]. Considering a fractionation (Δ) of −1.3‰  between the fuel source and the emitted CO_2_ [41], then the carbon isotope composition of the fuel used by the RTPPC is around −28.7‰.

## 4. Conclusions

The results of four-week monitoring campaign of particulate matter and gaseous pollutants, as well as meteorological parameters are presented. The present study was conducted in one of the most industrial energy-intense production corridors of Mexico, located 60 km north of Mexico City. The databases are also meant to be used as inputs for future modeling studies and to quantitatively determine the influence of emissions of this region on the air quality in the northern area of MCMA.

Early in the morning a shallow stable layer is formed, with light south-southwesterly winds. These conditions favor accumulation of air pollutants below 100 m and with a limited dispersion of pollutants towards the north of Tula. After midday, a well developed mixing layer is observed up to 1500 m, favoring the mixing of pollutants at higher altitudes. In addition, a superadiabatic layer near the surface, with a clear northern wind component, brings back the pollutants that were transported northward early in the morning. In the afternoon and evening, a similar vertical atmospheric profile is observed; a thicker mixing layer reaches in average 2500 to 3000 m, and a featuring weak low level jet responsible of transporting air pollutants at regional scales. According to the authors' knowledge, this is the first time this feature is reported for this region.

The results of the criteria pollutants concentration at TUL showed that during the study period, most of the pollutants were found to be below the standards, except for PM_10_ and SO_2_ that were above the limits twice and O_3_ once in each case. Nonetheless, the spatial distribution of these pollutants varies according to location and time of the day. The highest concentrations of O_3_ and SO_2_ were measured at the southeast of the refinery within a distance of more than 15 km. 

Elevated PM_10_ concentrations (178.6 *μ*g/m^3^) exceeding the standard measured at the urban-industrial site of JAS were associated to the increase of wind speeds up to 8.0 m/s. Additionally, this site was highly impacted by local limestone dust since the coarse fraction ranged from 40 to 79% with an average of 56%. Preliminary results from chemical analysis showed that high PM_10_ concentrations were driven by calcium mainly due limestone mining for cement materials and limestone quarries nearby JAS and in the surroundings of Tula, whilst in Mexico City, it is driven by silicon. PM_10_ levels at TEP showed an average of 75.1 *μ*g/m^3^, while the PM_2.5_ average was 25.7 *μ*g/m^3^, and an average PM_10_/PM_2.5_ mass ratio of 0.68 indicating that almost 70% of the PM_10_ are constituted by PM_2.5_. The above suggests that this site was influenced by higher emission from fossil fuel combustion processes from the refinery and the power plant, among other sources in the vicinity. 

PM_2.5_ concentrations were influenced by a peak recorded during the morning, similar to the continuous black carbon measurements, which agrees with a recurrent stable layer formed near the surface before 8:00 h and light winds favoring particle accumulation.

Average hourly aerosol light absorption was 22 Mm^−1^ which is smaller compared to both urban and rural sites. Average hourly aerosol scattering (76 Mm^−1^) was higher compared to a rural site but much lower than at Mexico City. Elevated values were related to uncommon flaring activities in the refinery in addition to changes in the boundary layer height. High scattering values were associated to a persistent shallow stable atmospheric layer which traps most primary pollutants emitted close to the surface. Low scattering values were related to the development of the mixing layer favoring the dilution of air pollutants.

Elevated BC concentrations were inversely coincident with the wind speed peak with light winds as the boundary layer is stable favoring stagnation of pollutants. In addition, the decrease of the 1/WS favored dilution of pollutants with the lowest levels of BC.

Stable carbon isotope measurements in the biomonitor species *Tillandsia recurvata* provide the long-term signature of pollutant dispersion in the region. Results show that the emission plume from the refinery and nearby thermoelectric power plant directly affects the SE sector of Mezquital Valley where the plume is directed by the prevalent winds and then constrained by a mountain range preventing its fast dispersion. Using stable isotope-mass balance, the carbon contribution as CO_2_ near the emission source to this epiphyte is estimated at around 5% of the total assimilated by the plant.

The population in this region is exposed to a multi-pollutant environment, including high levels of sulfur dioxide, submicrometric particles, and black carbon. In addition, frequent adverse meteorological conditions in the morning may exacerbate acute and chronic exposition to these pollutants. The influence of emissions from the RTPPC also affects, to a less extent, the population of MCMA's northern sector. The above-mentioned results evidence the need to establish a contingency plan to avoid population exposure to high concentrations of pollutants in addition to the reduction in industrial emissions to improve wellbeing. Studies within the area that determine the impacts of specific particulate constituents and sources are needed for the effective design of air quality control policies. Finally, a continuous monitoring of both criteria pollutants and meteorological parameters along the Tula-Vito-Apasco corridor is needed to support actions considered in the contingency plan and to better understand impacts of industrial activities on human and ecosystems health. 

## Figures and Tables

**Table 1:** Equipment deployed at each site and frequency of measurements.

Equipment	Site	Frequency
Rawinsondes	TUL	8, 12, 15, 18 h
Surface meteorology	TUL, JAS, TEP	6 min
Minivols (PM_2.5_, PM_10_)	JAS, TEP	24 hrs
Denuders	JAS, TEP	24 hrs
SMPS + DMA	JAS	15 min
Nephelometer	JAS	1 min
Aethalometer	JAS	1 min
Criteria pollutants	JAS	1 min
(CO, O_3_, NO_2_, SO_2_)	TEP	
MiniDOAS	Mobile	Continuous
Pilot balloons	TUL	1 hr from 10 to 18 h

SMPS: scanning mobility particle sizer; DMA: differential mobility analyzer.

**Table 2:** Average, maximum, and minimum of 24 h and 12 h of PM_10_ and PM_2.5_ during the sampling period of March 24–20 April, 2006.

	Jasso			Tepeji		
	Average	Maximum	Minimum	Average	Maximum	Minimum
PM_2.5_ 24 h	31.0	52.0	14.3	25.7	53.1	14.5
PM_2.5_ 06:00–18:00	45.5	70.3	18.2	35.5	75.1	16.8
PM_2.5_ 18:00–06:00	31.0	59.2	10.3	24.9	37.6	12.0
PM_10_ 24 h	75.1	178.6	30.4	36.8	53.2	22.4
